# Use of two posterior lip augmentation devices for recurrent total hip arthroplasty dislocation in select patients

**DOI:** 10.1308/003588413X13511609958055d

**Published:** 2013-03

**Authors:** D Yeung, K Rourke, N Pradhan

**Affiliations:** Warrington and Halton Hospitals NHS Foundation Trust, UK

A single posterior lip augmentation device (PLAD) can be used as a revision technique for a recurrent posteriorly dislocating total hip arthroplasty with satisfactory results.^1^ Recurrent dislocating hips with soft tissue laxity not controlled with a single posterior–lateral PLAD is treated traditionally with a constraint cup. We advocate the use of a PLAD placed in the anterior–medial aspect alongside a posterior–lateral device to achieve the same result. We have experience of three patients thus treated, with satisfactory results.
